# Inhibited neural response during interpersonal conflict: insights from fNIRS hyperscanning

**DOI:** 10.3389/fpsyg.2025.1712278

**Published:** 2025-11-12

**Authors:** Kang Cao, Mingming Zhang, Yuxuan Zhang, Jie Li

**Affiliations:** 1 School of Psychology, Inner Mongolia Normal University, Hohhot, China; 2 School of Arts and Education, Chizhou University, Chizhou, China; 3 School of Psychology, Shanghai Normal University, Shanghai, China; 4 School of Management, Tianjin University of Traditional Chinese Medicine, Tianjin, China

**Keywords:** interpersonal conflict, functional near-infrared spectroscopy (fNIRS), hyperscanning, inter-brain synchronization, social interactions

## Abstract

Interpersonal conflict is a core yet complicated part of social interaction, involving complex mental and emotional processes. However, the neural mechanisms underlying interpersonal conflict are still not fully understood. This study employed functional near-infrared spectroscopy (fNIRS) hyperscanning to explore the brain activity related to interpersonal conflict through both passive video viewing and active role-playing paradigms. The results revealed an unexpected activation pattern – brain activity was highest at rest, lower during conflict, and lowest during neutral interactions (i.e., rest > conflict > neutral) in all ROIs except the rTPJ during active role-playing. This indicates a cortical deactivation effect when people engage in social processing. Additionally, the study found that inter-brain synchronization (IBS) between the two participants' brains decreased significantly during conflict compared to non-conflict conditions. These findings provide neurocognitive evidence for disrupted interpersonal alignment during conflict and highlight potential intervention targets—such as perspective-taking and interpersonal attunement—for enhancing social functioning in challenging interactions.

## 1 Introduction

Interpersonal interactions are crucial to human social life, shaping various social relationships that greatly impact individuals' psychological and emotional health. However, maintaining these relationships naturally requires managing conflicts, which inevitably emerge from incompatible goals, differing interests, or conflicting values values (Donohue and Cai, 2014). Interpersonal conflict occurs when an individual's actions obstruct, interfere with, or adversely affect another person's goals or behaviors, thereby reducing mutual effectiveness and cohesion (Burton et al., 1972). Such conflictual interactions usually manifest through dynamic exchanges involving ongoing verbal and nonverbal communication, as well as mutual inferences about each other's intentions, emotions, and thoughts. Because of their widespread presence and significant impact, understanding the neurocognitive mechanisms behind interpersonal conflict is crucial not only for advancing theory but also for practical use in conflict resolution and psychological treatment (Flanagan et al., 2019).

Despite extensive psychological research on interpersonal conflict, current empirical methods face significant limitations. Many studies rely on retrospective self-report surveys or categorize incidents into broad types (e.g., task vs. relationship conflict; Wright et al., 2017; Hill et al., 2019). This approach is limited because it offers only a static, subjective snapshot of conflict intensity or type, rather than capturing the dynamic interaction as it happens. In fact, many existing conflict scales were developed years ago and have not adapted to the broader understanding of interpersonal conflict, which now encompasses a wider range of behaviors, thoughts, and emotions *(*DeBaylo and Michel, 2022). By reducing conflict to broad categories and overall intensity ratings, researchers risk overlooking the complex, immediate processes that define real conflicts. Interpersonal conflict is inherently dynamic and interactive, involving a series of actions and reactions between dependent parties (Au and Lam, 2017; Long et al., 2021). It has been described as “a dynamic process” where parties experience negative emotional responses to perceived disagreements and interference with their goals (Barki and Hartwick, 2004; Ejbye-Ernst et al., 2022). However, typical empirical methods rarely observe these live dynamics. Hyperscanning provides such a framework by enabling the simultaneous measurement of neural activity in interacting partners (Balconi and Vanutelli, 2017; Nam et al., 2020).

An expanding body of hyperscanning research has demonstrated notable inter-brain synchronization (IBS) during cooperative interactions, indicating shared cognitive states and emotional alignment among participants (Liu et al., 2017; Balconi et al., 2021). While increased IBS during cooperation has been interpreted as a neural marker of shared intentionality and mutual understanding, it remains unclear whether and how such coupling deteriorates when interactions become adversarial. Besides, the neural synchronization patterns that occur during conflictual interactions are relatively underexplored, which limits our understanding of how neural coupling may fundamentally differ when interpersonal interactions shift from cooperative to adversarial contexts (Long et al., 2021; Ryu and Kim, 2024). Among available hyperscanning modalities, functional near-infrared spectroscopy (fNIRS) offers a unique balance between ecological validity and neural specificity. Although it provides lower temporal resolution than electroencephalography (EEG) and reduced spatial resolution compared to functional magnetic resonance imaging (fMRI) (Cui et al., 2011; Chiarelli et al., 2017), fNIRS enables participants to engage in naturalistic face-to-face interactions with minimal physical constraints—an essential feature for capturing the dynamics of interpersonal conflict.

Recent findings from hyperscanning studies indicate a decrease in IBS during contexts involving interpersonal conflict. This decline may be due to disruptions in shared attentional states and individual emotional regulation processes. For example, Liang et al. observed reduced IBS during tasks involving interpersonal conflict compared to resting states (Liang et al., 2022), especially in brain regions related to emotion regulation and social cognition, such as the dorsolateral prefrontal cortex (DLPFC), inferior frontal gyrus (IFG), and temporoparietal junction (TPJ). These decreases may reflect a shift toward self-focused cognitive and emotional processing, which reduces the neural coupling usually seen during active social interactions. Similarly, Hirsch and colleagues found that scenarios involving disagreement were also linked to lower IBS compared to agreement scenarios, further supporting the idea that divergent perspectives interfere with shared cognitive states and decrease inter-brain coupling (Hirsch et al., 2021). However, further replication and detailed investigation of this phenomenon across different task paradigms are necessary.

Employing multiple paradigms allows for a more thorough exploration of the psychological and neural processes involved in interpersonal conflict. The video-viewing paradigm provides a highly controlled form of experimental stimulus. All participants view the same standardized conflict scenes, which minimizes extraneous variability caused by stimulus differences and improves the comparability and reproducibility of results (Ryu and Kim, 2024). Conversely, the role-playing paradigm emphasizes ecological validity by involving participants in face-to-face scripted conflict interactions (Redcay and Schilbach, 2019). This dyadic, interactive approach more closely resembles real-life social exchanges and elicits spontaneous emotional and behavioral reactions. Participants must actively engage in verbal and nonverbal communication, demanding more intensive cognitive and emotional effort. This engagement enables researchers to observe dynamic processes such as emotion regulation and interpersonal synchrony. By using both paradigms, the current study can determine whether IBS and emotional responses differ depending on whether individuals are observing or participating in conflict, thus increasing the generalizability of the findings (Guo et al., 2022). Additionally, this dual-paradigm design balances internal validity and ecological validity: the video paradigm offers consistent, tightly controlled stimuli, while the role-play paradigm enhances the real-world relevance of the results.

The current study focused on four main brain regions that are key to emotional regulation, cognitive control, and social cognition: the left IFG (lIFG), bilateral DLPFC (lDLPFC, rDLPFC), and the right TPJ (rTPJ). The IFG and DLPFC are essential areas involved in managing emotions and exercising cognitive control, with strong evidence showing their roles in influencing affective responses and exerting inhibitory control during emotionally intense social interactions(Gross, 2015). The rTPJ has consistently been associated with social cognitive functions such as perspective-taking and mental state attribution (Frith et al., 2006). As a result, disruptions in shared attention or impaired perspective-taking, which are common in conflict situations, are expected to lead to decreased IBS within these critical regions. Ultimately, this study aims to deepen a comprehensive and detailed understanding of the neural and psychological dynamics of interpersonal conflict by integrating multiple paradigms. Based on limited prior research and social neuroscience frameworks (Zaki and Ochsner, 2012), we hypothesize that interpersonal conflict will reliably cause reduced IBS compared to neutral and resting conditions, indicating diminished shared emotional attunement.

## 2 Experiment 1: passive interpersonal conflict paradigm

### 2.1 Materials and methods

#### 2.1.1 Participants

A total of 106 right-handed undergraduate students (22 males and 84 females), aged between 18 and 21 years (*M* = 19.54, *SD* = 1.79), participated in the experiment. Participants were randomly assigned to 53 same-gender dyads, with each pair consisting of individuals who were acquaintances. All participants possessed normal or corrected-to-normal vision and had no history of neurological or psychiatric disorders. The sample size of *N* = 28 was determined through a power analysis conducted using G^*^Power 3.1, with an α level of 0.05 and a power of 0.8 to detect a medium effect size (*f* = 0.25). This power analysis was consistently applied across all experiments reported in this article. Before participating, each individual provided written informed consent. Upon completing the experiment, participants received a show-up fee of 15 yuan. This study was approved by the Research Ethics Board of the School of Arts and Education at Chizhou University (Approval No. 2022030302) and was conducted in accordance with the Declaration of Helsinki.

#### 2.1.2 Experimental tasks and procedure

Four standardized videos were produced, each featuring two actors participating in scripted dialogues. These included three scenarios showing interpersonal conflict and one neutral control scenario, with each video lasting 60 secs. Gender-matched dyads were assigned to watch matching videos (e.g., female–female dyads viewed videos with two female actors). The videos were shot in a controlled laboratory setting to ensure uniform context, and the actors rehearsed their scripts thoroughly to keep performance quality consistent.

**Figure 1:**
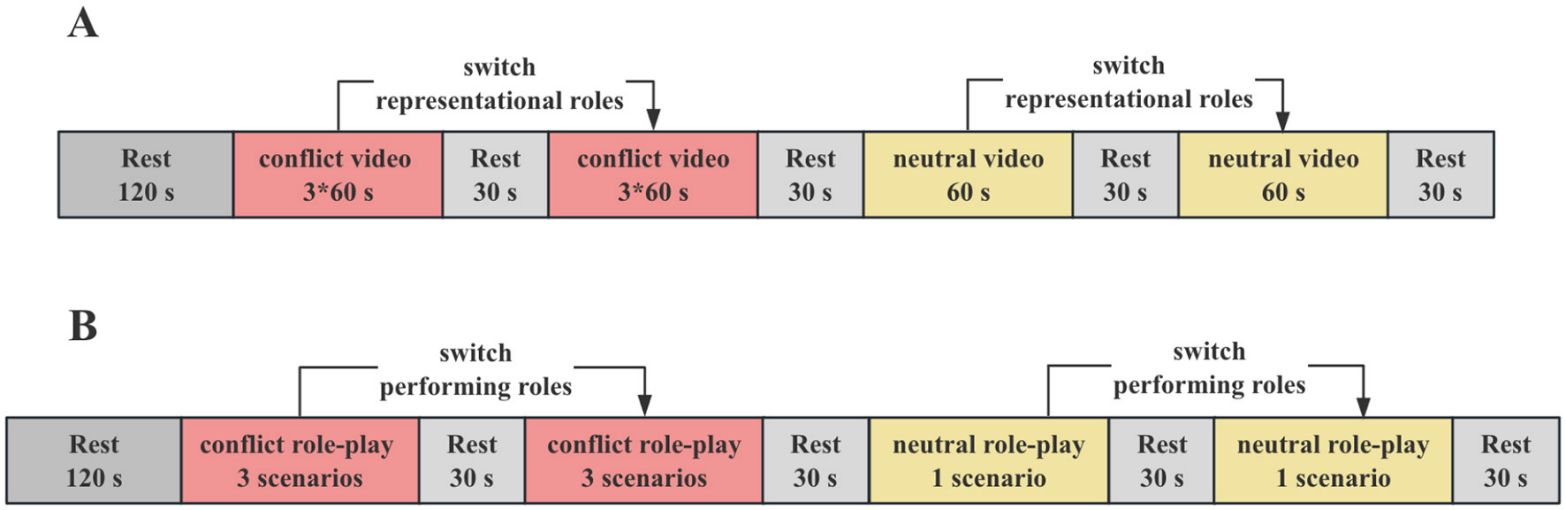
Experimental design. **(A)** Experimental 1 procedure and entire timeline; **(B)** Experimental 2 procedure and entire timeline.

Participants sat next to each other, facing a screen. After placing the fNIRS cap and locating the optodes, the session started with a 120 sec rest period. The dyads then watched three conflict-related videos, shown in a random order. Next, participants swapped roles and viewed the same scenarios again, but in a different random order. A neutral video was shown twice in a row afterward. Each video was preceded by a 30 sec rest to allow hemodynamic signals to reset to baseline. This process resulted in eight viewing trials per dyad: six conflict trials and two neutral trials. The full experimental procedure is shown in Figure 1A.

Before the video task, participants completed a series of affective and social evaluation measures to determine if the conflict videos successfully evoked the intended emotional and social responses, serving as a validity check for the conflict paradigm: (1) perceived partner adorableness (rated on a 9-point scale, 1 = extremely unadorable, 9 = extremely adorable), (2) current mood valence (1 = extremely unhappy, 9 = extremely happy), and (3) the intended allocation of a ¥100 participation reward to their partner (range: ¥1–¥100). The same measures were taken after the task to assess changes in affective state and social perception. Additionally, participants evaluated (4) how much they identified with the assigned role during video viewing (1 = not at all, 9 = completely), and (5) the perceived congruence between the two characters' behavioral goals (1 = extremely incongruent, 9 = completely congruent).

### 2.2 fNIRS data acquisition

**Figure 2:**
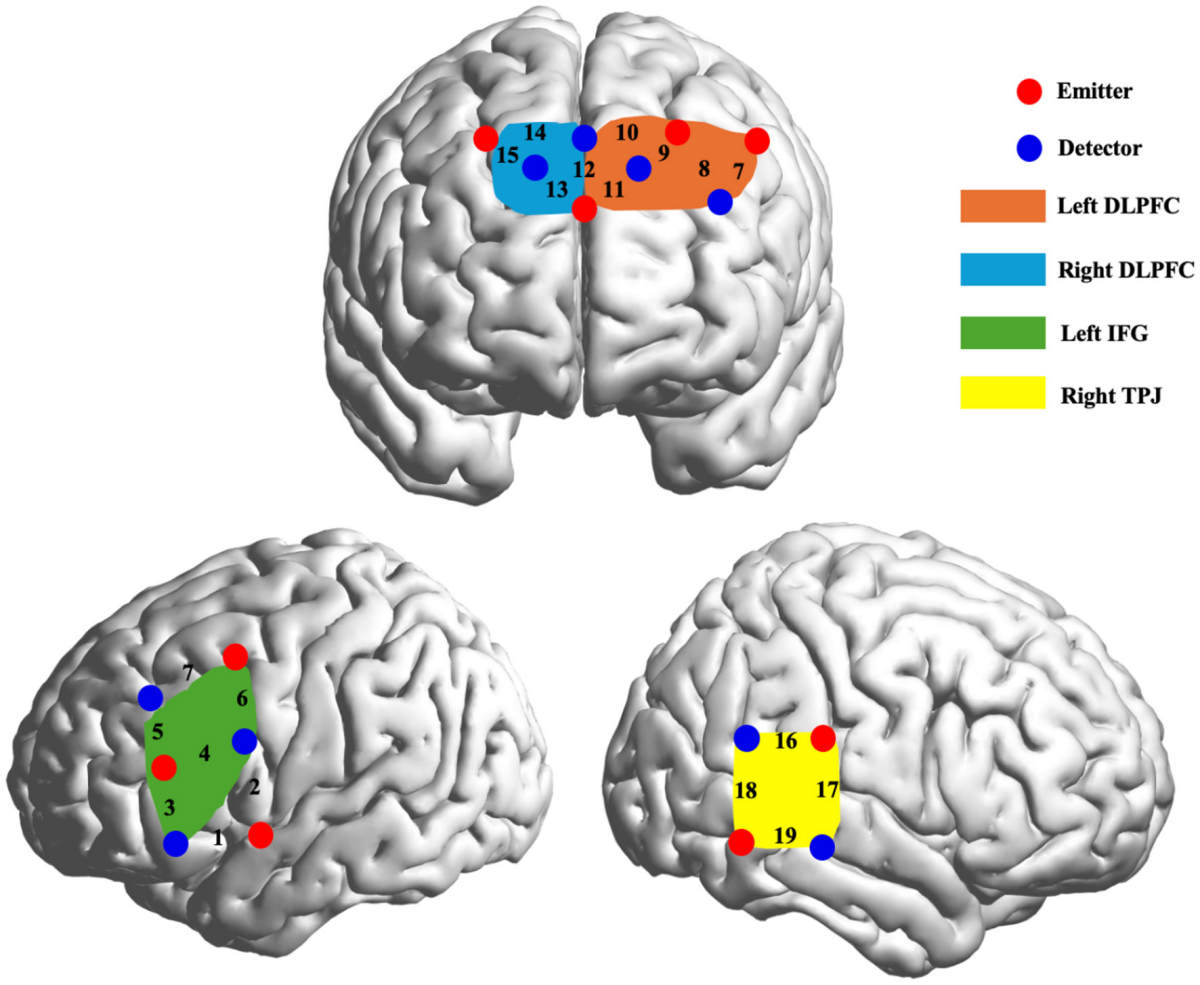
Optode probe set and channel layout. The number refers to the channel number and indicates its position. The ROI grouping was as follows: (1) Left inferior frontal gyrus (IFG): channels 3, 4, 5 and 6; (2) Left Dorsolateral Prefrontal Cortex (DLPFC): channels 7, 8, 9, 10 and 11; (3) Right DLPFC: channel 12, 13, 14, 15; (4) Right temporoparietal junction area (TPJ): channels 16, 17, 18 and 19.

The fNIRS data were collected using the NIRSport2 system (NIRx Medical Technologies) at a sampling rate of 7.8125 Hz, with wavelengths of 760 nm and 850 nm. Data collection involved 19 channels with eight emitters and eight detectors, arranged in 6 × 6 and 2 × 2 arrays with an inter-optode distance of 30 mm. Optode placement followed the international 10–20 system, with the central column aligned along the sagittal plane and the lowest row along the axial plane. Anatomical locations were digitally registered using a Polhemus Fastrak 3D Digitizer, and the coordinates were later transformed to Montreal Neurological Institute (MNI) space using the NIRS_SPM MATLAB package (Table 1). The analysis of channels 1 and 2 is temporarily excluded due to their anatomical location. The remaining 17 channels were grouped into four brain regions of interest (ROIs): lDLPFC, rDLPFC, lIFG, and rTPJ (Figure 2).

**Table 1:** The MNI coordinates and probabilistic cortical localization of all 19 channels.

**Channels**	**MNI coordinates**			**Brodmann' s area**	***p***
	***x***	***y***	***z***		
1	−56	16	−14	38 - Temporopolar area	0.98
2	−62	8	5	48 - Retrosubicular area	0.62
3	−55	38	−1	45 - pars triangularis Broca's area	0.76
4	−58	26	16	45 - pars triangularis Broca's area	0.76
5	−47	43	24	45 - pars triangularis Broca's area	0.83
6	−56	14	35	44 - pars opercularis	0.67
7	45	31	42	9 - Dorsolateral prefrontal cortex	0.41
8	−30	46	42	9 - Dorsolateral prefrontal cortex	0.82
9	−13	53	45	9 - Dorsolateral prefrontal cortex	1
10	−10	46	52	9 - Dorsolateral prefrontal cortex	0.76
11	−4	62	36	9 - Dorsolateral prefrontal cortex	0.5
12	1	55	41	9 - Dorsolateral prefrontal cortex	0.93
13	9	62	36	9 - Dorsolateral prefrontal cortex	0.56
14	13	46	53	9 - Dorsolateral prefrontal cortex	0.74
15	17	52	46	9 - Dorsolateral prefrontal cortex	1
16	70	−35	29	40 - Supramarginal gyrus part of Wernicke's area	0.41
17	73	−21	8	22 - Superior Temporal Gyrus	0.77
18	69	−50	11	22 - Superior Temporal Gyrus	0.5
19	72	−37	−10	21 - Middle Temporal gyrus	0.52

After converting MNI coordinates to Talairach space (Laird et al., 2010), we identified the corresponding Brodmann areas. A single channel might cover several brain regions, but the combined overlap never surpassed 100%. We reported only the channels with the highest or near-highest overlap.

### 2.3 Data analysis

The fNIRS data were collected using the NIRSport2 system (NIRx Medical Technologies) at a sampling rate of 7.8125 Hz, with wavelengths of 760 nm and 850 nm. Data collection involved 19 channels. Participant-level preprocessing was performed following established hyperscanning methods. The raw fNIRS data for each participant were processed with the HOMER2 MATLAB package (Huppert et al., 2009). First, the quality of the fNIRS signals was evaluated using the *enPruneChannels* function. Channels with a coefficient of variation (CV) greater than 15% were considered unreliable and excluded. If more than 50% of the channels were classified as unreliable, the data for the entire dyad were excluded from further analysis. However, based on these criteria, no dyads were excluded in this study. The raw fNIRS data were then transformed into optical density (OD) data using the *hmrIntensity2OD* function. Motion artifacts within the OD data were detected using the *hmrMotionArtifactByChannel* function, with parameters set to *tMotion* = 0.5, *tMask* = 3, *STDEVthresh* = 10, and *AMPthresh* = 50. These artifacts were later corrected using the wavelet-based motion artifact removal method via the *hmrMotionCorrectWavelet* function. The bandpass filtering was applied with the *hmrBandpassFilt* function, with high-pass and low-pass filter cutoffs set at 0.01 and 0.07, respectively. Finally, using the modified Beer–Lambert law, the OD data were transformed into concentrations of oxyhemoglobin (HbO) and deoxyhemoglobin (HbR) via the *hmrOD2Conc* function. Due to the high signal-to-noise ratio of the HbO signal, our analysis in this study focused solely on the HbO signal (Cui et al., 2012).

Intra-brain activation was examined at the individual level. For each ROI, the HbO values of the channels corresponding to the specific ROI, based on their anatomical locations, were averaged for each participant. After a Fisher *z*-transformation, a series of one-way repeated measures ANOVAs was performed on the ROI data across different experimental conditions. To control for multiple comparisons, a false discovery rate (FDR) correction was applied, with a significance threshold of *p* < 0.05.

**Figure 3:**
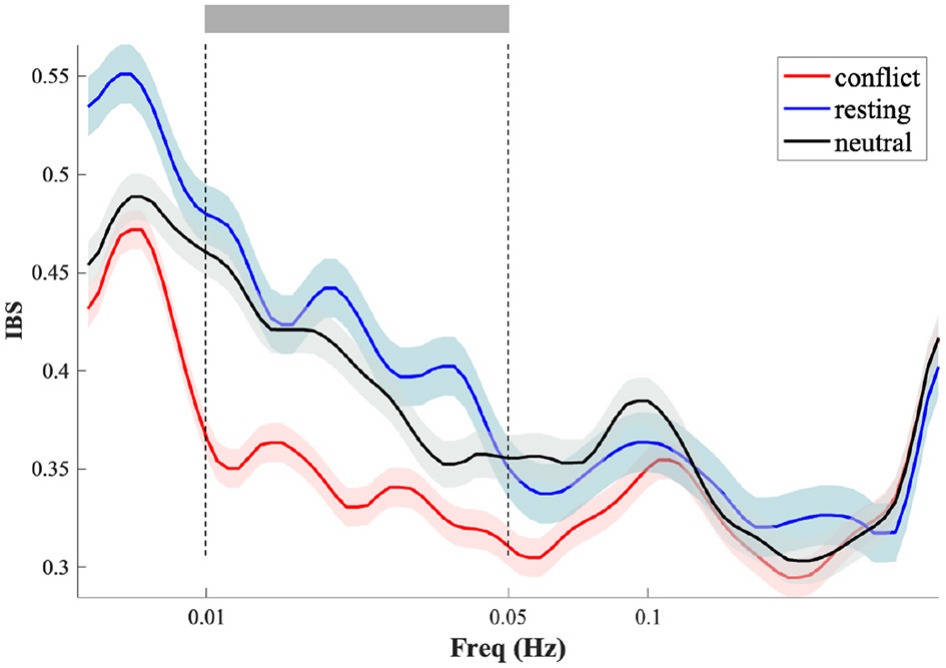
IBS Variations when identifying the frequency band of interest. Significant frequency from 0.01 to 0.05Hz (20–100s) under Experiment 1. The interval delineated by two vertical dashed lines shows the significant frequency range.

The IBS at the dyadic level was analyzed by measuring the synchronization between participants' HbO time series within each dyad using the wavelet transform coherence (WTC) MATLAB package (Grinsted et al., 2004). This method was chosen to assess the temporal relationship of HbO signals between pairs of participants, a technique widely used in previous research (Cui et al., 2012; Zhang et al., 2021a). Next, a data-driven frequency-band selection approach was applied to compare wavelet coherence values across two task conditions and a resting phase (Zhang et al., 2021b). For each condition, WTC was calculated across all frequency bands to determine the average coherence during the task. Then, one-way repeated measures ANOVAs were performed on these average WTC values across the two task conditions and resting phase to identify a statistically significant frequency band (0.01–0.05 Hz, Figure 3). This frequency band was chosen to exclude high-frequency and low-frequency noise, such as physiological signals related to blood pressure (about 0.1 Hz), respiration (about 0.2–0.3 Hz), and heart rate (1 Hz) (Nozawa et al., 2016; Zhang et al., 2020). The IBS within this selected frequency band was then averaged across conditions (Zhao et al., 2023). Following this, the IBS for each pair of channels within a specific ROI was averaged, and WTC values were computed and transformed using Fisher z-statistics (Chang and Glover, 2010). Additional one-way repeated measures ANOVAs were conducted on the task-related IBS across all frequencies of interest (FOIs) and ROIs, with condition (conflict, neutral, resting) as the within-participant factor. An FDR correction was also applied. Visualization was performed using the *BrainNet Viewer* MATLAB package (Xia et al., 2013).

### 2.4 Results

#### 2.4.1 Affective and social evaluations

To evaluate the effect of the conflict videos on participants' social and emotional assessments, paired-sample *t*-tests were performed comparing scores before and after viewing. Change scores (*M*_*D*_) were calculated as the mean before viewing minus the mean after viewing. Perceived partner adorableness significantly decreased following the video task, *M*_*D*_ ± *SE* = 0.86 ± 0.18, *t* (105) = 4.83, *p* < 0.001, Cohen's *d* = 0.47. Self-reported happiness also significantly declined, *M*_*D*_ ± *SE* = 0.85 ± 0.17, *t* (105) = 4.93, *p* < 0.001, Cohen's *d* = 0.48. The amount of reward given to the partner also significantly decreased, *M*_*D*_ ± *SE* = 2.99 ± 1.39, *t* (105) = 2.16, *p* =0.03, Cohen's *d* = 0.21. To examine participants' engagement with the video task, one-sample *t*-tests were conducted comparing the average ratings to the scale midpoint (expected value = 5). Participants reported high engagement when viewing neutral videos, *M* = 6.10, *t* (105) = 7.99, *p* < 0.001, Cohen's *d* = 0.77. They also demonstrated moderate engagement with conflict videos (*M* = 5.51, *t*(105) = 3.61, *p* < 0.001, Cohen's *d* = 0.35) and perceived low goal congruence between the characters in the conflict videos (*M* = 3.21, *t* (106) = −15.96, *p* < 0.001, Cohen's *d* = 1.54).

#### 2.4.2 Intra-brain activation

**Figure 4:**
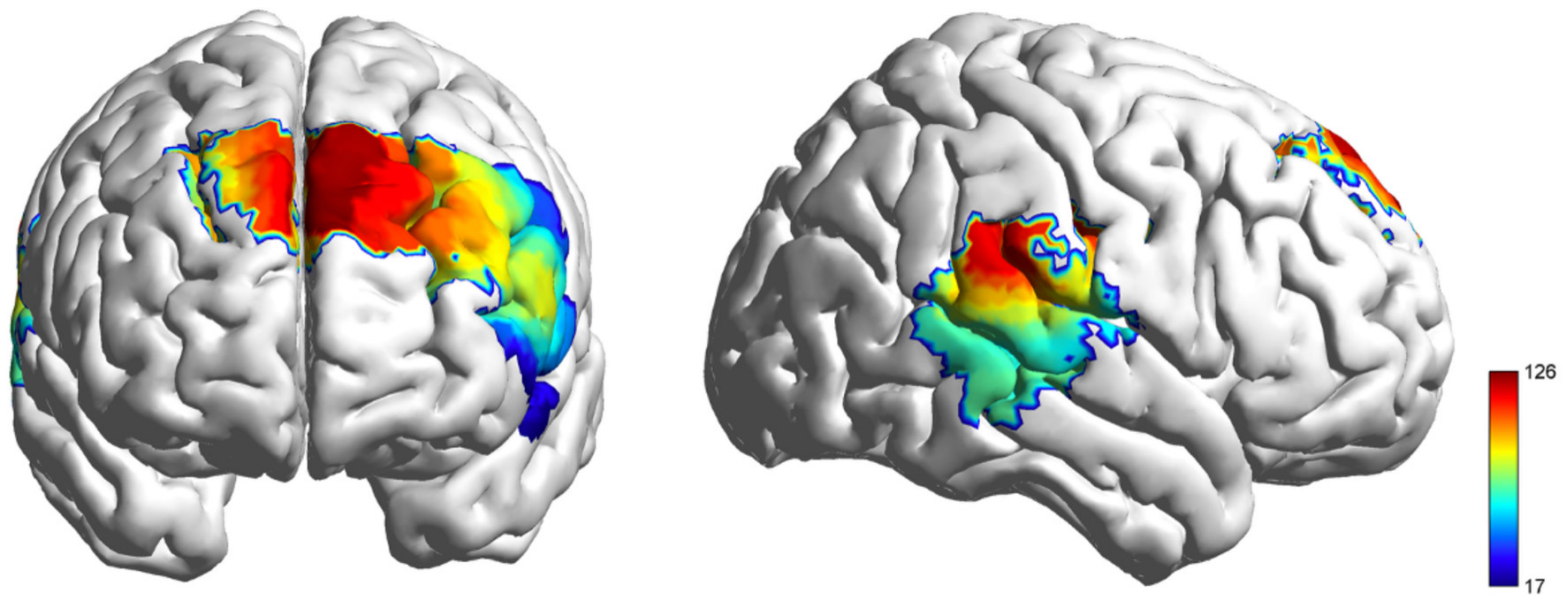
HbO activation F-test maps generated from individual fNIRS channels, illustrating cortical hemodynamic responses during the video viewing.

**Figure 5:**
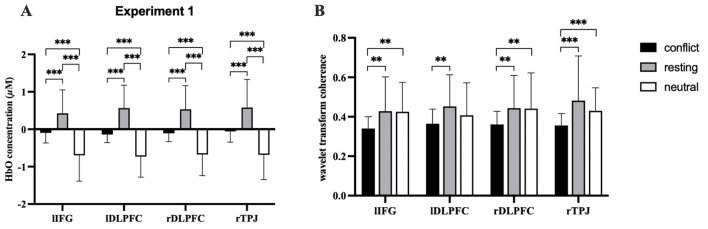
Results of the Analysis. **(A)** Differences in the level of brain activation of all ROIs. **(B)** Differences in IBS of all ROIs. The error bars represent the standard deviation (SD*)*. *p <0.05.**p <0.01.***p <0.001.

Significant differences appeared in intra-brain HbO across conditions in all ROIs (lIFG: *F*_(2, 210)_ = 80.828, FDR-corrected *p* < 0.001, $\eta_{p}^{2}$ = 0.435; lDLPFC: *F*_(2, 210)_ = 143.312, FDR-corrected *p* < 0.001, $\eta_{p}^{2}$ = 0.577; rDLPFC: *F*_(2, 210)_ = 112.502, FDR-corrected *p* < 0.001, $\eta_{p}^{2}$ = 0.517; and rTPJ: *F*_(2, 210)_ = 97.374, FDR-corrected *p* < 0.001, $\eta_{p}^{2}$ = 0.481; Figure 4). Critically, resting periods exhibited positive HbO activation, while both conflict and neutral conditions had negative HbO responses (deactivations). Post-hoc tests revealed higher HbO during resting compared to conflict conditions, with conflict conditions exceeding neutral conditions (Figure 5A).

#### 2.4.3 Inter-brain coupling

**Figure 6:**
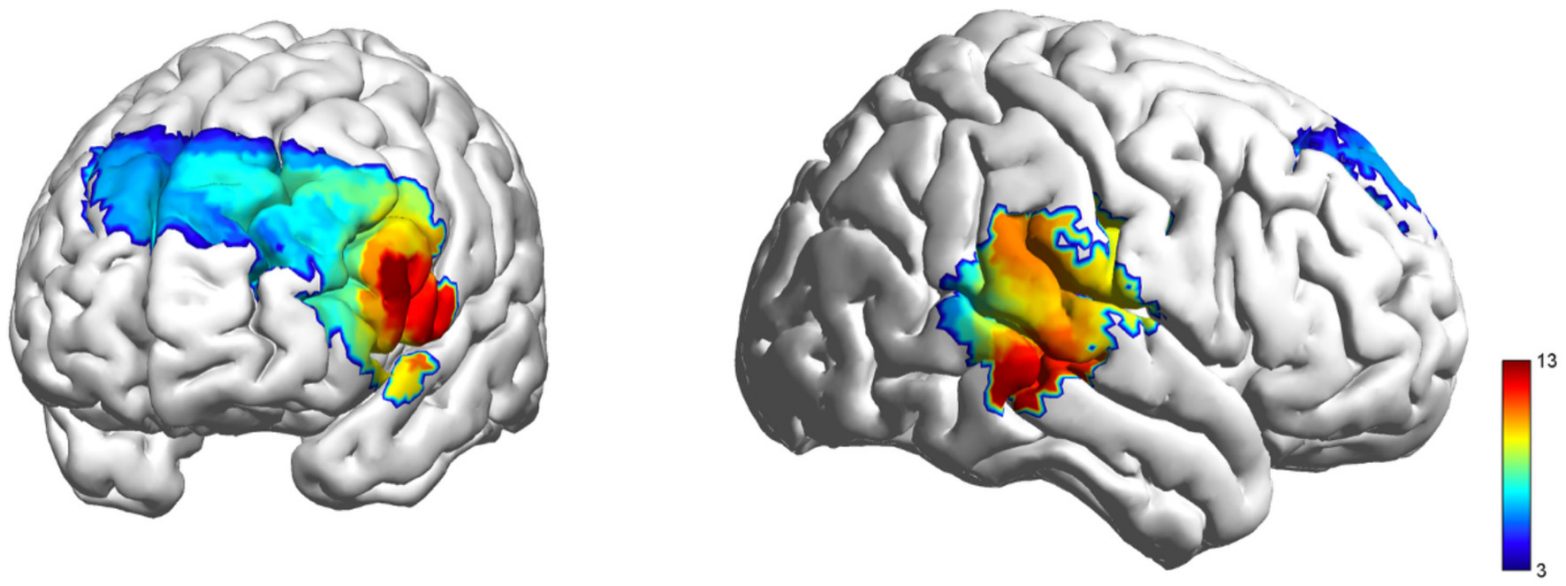
Coherence F-test maps generated from the dyad's fNIRS channels, illustrating inter-brain synchronization during the video viewing.

For IBS, significant differences between conditions were observed in lIFG: *F*_(2, 104)_ = 6.968, FDR-corrected *p* < 0.01, $\eta_{p}^{2}$ = 0.118; lDLPFC: *F*_(2, 104)_ = 6.716, FDR-corrected *p* < 0.01, $\eta_{p}^{2}$ = 0.118; rDLPFC: *F*_(2, 104)_ = 5.767, FDR-corrected *p* < 0.01, $\eta_{p}^{2}$ = 0.100; and rTPJ: *F*_(2, 104)_ = 9.688, FDR-corrected *p* < 0.001, $\eta_{p}^{2}$ = 0.157 (Figure 6). Subsequent *post-hoc* tests revealed that IBS significantly decreased during conflict compared to neutral and resting conditions in lIFG, rDLPFC, and rTPJ; however, the reduction in IBS in rDLPFC during conflict vs. neutral conditions was not significant (Figure 5B).

## 3 Experiment 2: active interpersonal conflict paradigm

To assess how well the inter-brain synchronization patterns found in Experiment 1 could apply to situations with active participation, Experiment 2 used a role-playing setup. In this setup, participants performed scripted scenarios involving interpersonal conflicts and neutral conversations.

### 3.1 Participants and procedure

Fifty-eight right-handed undergraduates (10 males, 48 females; age range: 18–21 years, *M* = 19.72, *SD* = 1.67) formed 29 same-gender acquainted dyads. The procedures for consent and ethical approval were the same as in Experiment 1 (Figure 1B).

In Experiment 2, a structured role-playing format was used, consisting of three conflict scenarios and one neutral interaction. Participants were asked to memorize and perform scripts that matched their gender, actively engaging in both dialogues and gestures. The procedure was similar to that of Experiment 1, beginning with an initial rest period, followed by randomized enactments of conflict scenarios, role reversals with repeated performances, and subsequent neutral interactions. Each session was broken up by 30 sec rest periods, ending with a total of eight dialogues (six conflict and two neutral). As in Experiment 1, participants completed the same affective and social evaluation measures before and after the task. In addition, the configuration of the fNIRS, the placement of optodes, and the analytical protocols—including preprocessing, HbO activity calculation, IBS calculation, frequency selection, and statistical analysis—were replicated exactly from Experiment 1, thereby ensuring methodological consistency across the experiments.

### 3.2 Results

#### 3.2.1 Affective and social evaluations

Perceived partner adorableness significantly declined after the role-playing task, *t* (57) = 6.09, *p* < 0.01, Cohen's *d* = 0.77. Self-reported happiness also saw a significant drop, *t* (57) = 6.01, *p* < 0.01, Cohen's *d* = 0.76. The amount of reward given to the partner significantly decreased as well, *t* (57) = 5.29, *p* < 0.01, Cohen's *d* = 0.67. Participants reported high engagement with their assigned roles, *M* = 6.94, *t* (57) = 11.26, *p* < 0.01, Cohen's *d* = 1.43. They perceived low goal congruence between the two characters in the conflict scenarios they role-played, *M* = 3.15, *t* (57) = −12.94, *p* < 0.01, Cohen's *d* = 1.64.

#### 3.2.2 Intra-brain activation

**Figure 7:**
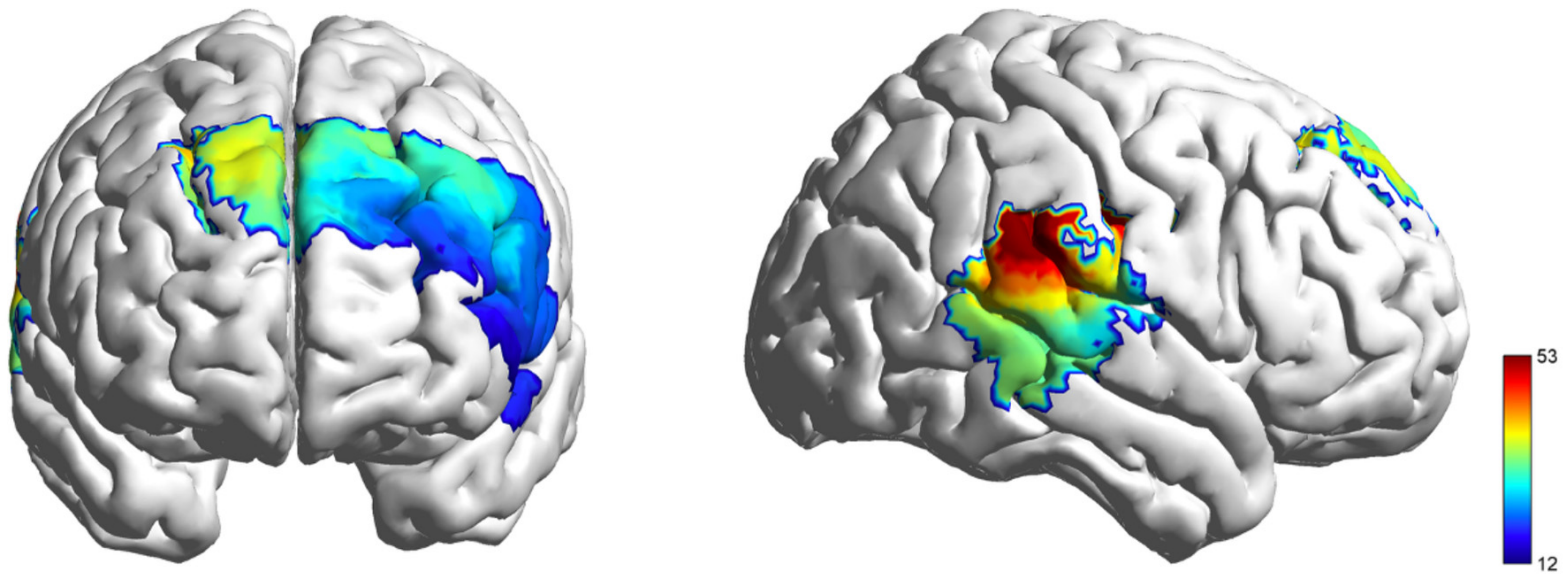
HbO activation F-test maps generated from individual fNIRS channels, illustrating cortical hemodynamic responses during the role playing.

**Figure 8:**
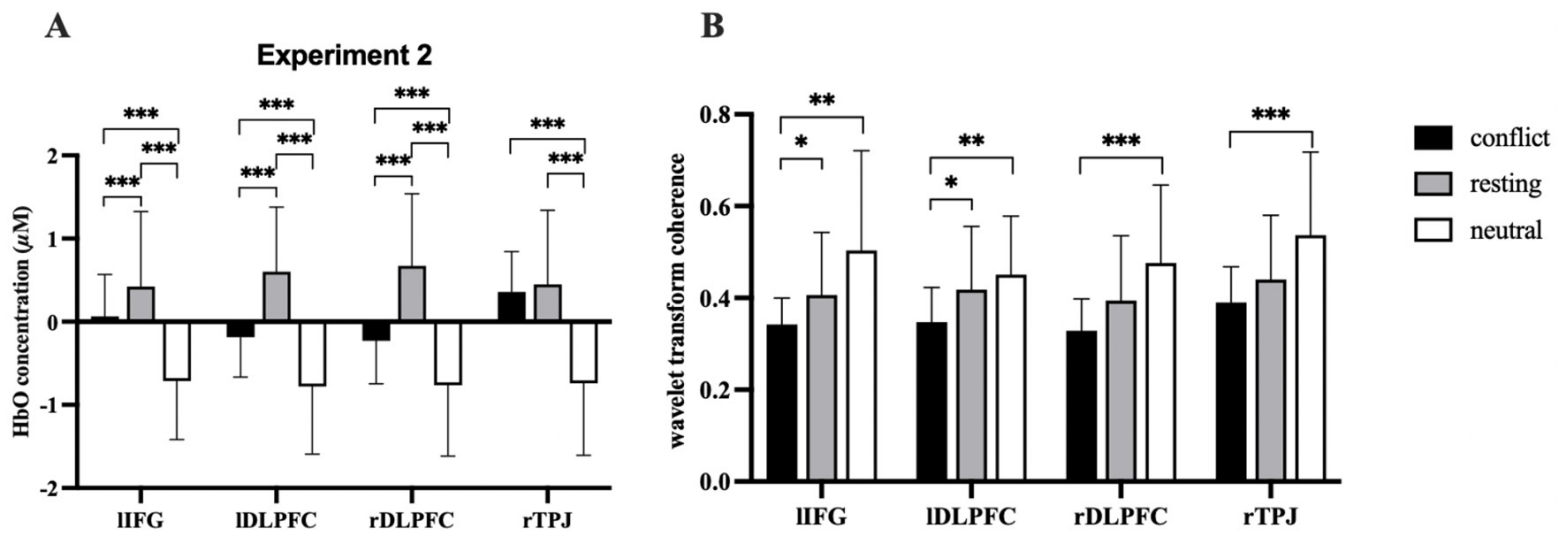
Results of the Analysis. **(A)** Differences in the level of brain activation of all ROIs during role-playing. **(B)** Differences in IBS across all ROIs during role-playing. The error bars represent the standard deviation *(SD)*. *p < 0.05,**p < 0.01,***p < 0.001 indicate levels of statistical significance.

ROI-specific HbO differences across conditions were significant (lIFG: *F* (2, 114) = 31.011, FDR-corrected *p* < 0.001, $\eta_{p}^{2}$ = 0.352; lDLPFC: *F*_(2, 114)_ = 49.767, FDR-corrected *p* < 0.001, $\eta_{p}^{2}$ = 0.466; rDLPFC: *F*_(2, 114)_ = 49.667, FDR-corrected *p* < 0.001, $\eta_{p}^{2}$ = 0.466; and rTPJ: *F*_(2, 114)_ = 43.007, FDR-corrected *p* < 0.001, $\eta_{p}^{2}$ = 0.430; Figure 7). In contrast to the findings of Experiment 1, the conflict condition demonstrated positive HbO activation in rTPJ, and the observed differences between the rest and conflict conditions did not achieve statistical significance. Apart from this, the results in other brain regions were consistent with those of Experiment 1 (Figure 8A).

#### 3.2.3 Inter-brain coupling

**Figure 9:**
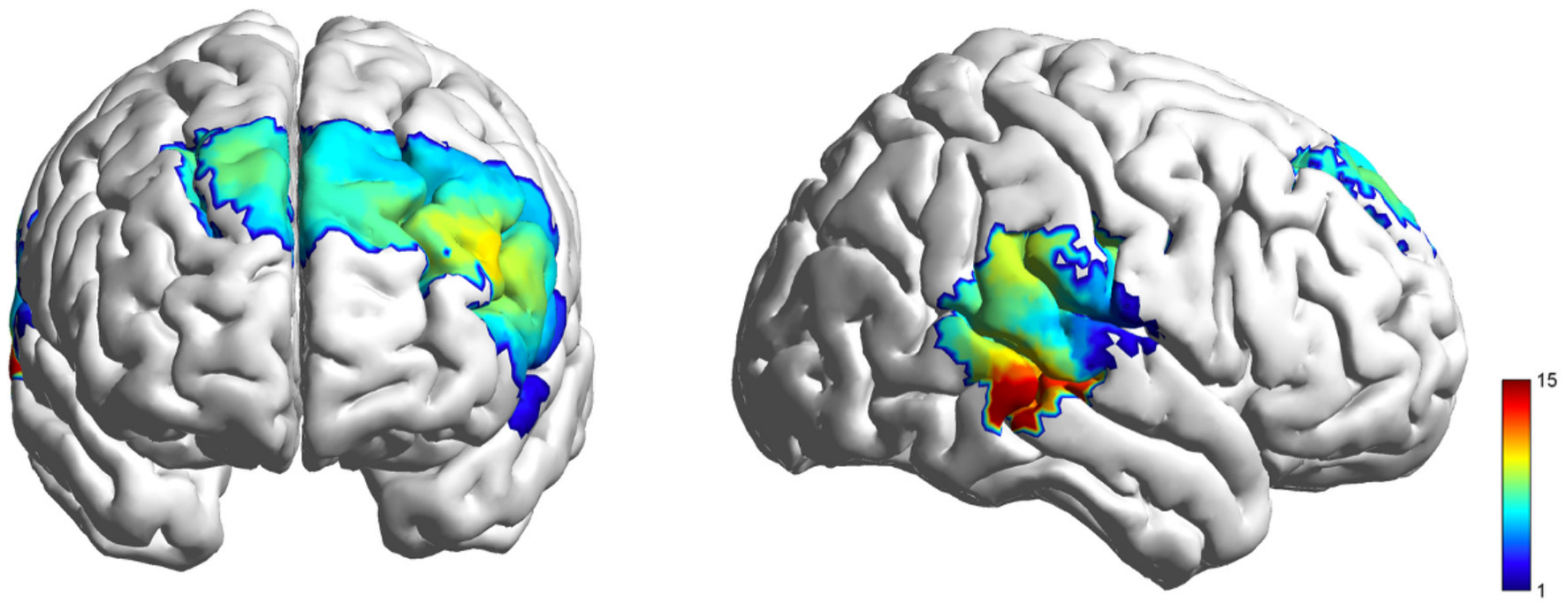
Coherence F-test maps generated for the dyad's fNIRS channels, illustrating inter-brain synchronization during the role playing.

IBS condition effects were significant for lIFG: *F*_(2, 56)_ = 7.318, FDR-corrected *p* < 0.01, $\eta_{p}^{2}$ = 0.207; lDLPFC: *F*_(2, 56)_ = 8.313, FDR-corrected *p* < 0.01, $\eta_{p}^{2}$ = 0.229; rDLPFC: *F*_(2, 56)_ = 8.345, FDR-corrected *p* < 0.01, $\eta_{p}^{2}$ = 0.230; and rTPJ: *F* (2, 56) = 9.879, FDR-corrected *p* < 0.001, $\eta_{p}^{2}$ = 0.261 (Figure 9). Subsequent post-hoc analyses indicated that within the lIFG and lDLPFC, the IBS showed a significant decrease when viewing conflict videos compared to both neutral videos and the resting phase. Conversely, in the rDLPFC and rTPJ, IBS during conflict video viewing was significantly reduced compared to neutral video viewing, yet it did not differ considerably from the resting phase (Figure 8B).

## 4 Discussion

This study aimed to explore how the brain functions during interpersonal conflict by using fNIRS hyperscanning during passive observation and active role-playing. It compared brain activation and inter-brain synchronization patterns. Results showed a unique activation pattern: brain activity increased above baseline during rest, while both conflict and neutral situations caused deactivation compared to rest, with neutral conditions showing even more deactivation than conflict (rest > conflict > neutral). Notably, the rTPJ was different from this pattern during active role-playing, showing a tendency toward positive activation in conflict scenarios. This suggests brain deactivation during social interaction. Additionally, IBS significantly dropped during conflict compared to non-conflict situations, indicating that neural coordination between partners was reduced during conflict. In simple terms, during conflict, the two brains were less “in tune” with each other than when they interacted calmly.

Firstly, the positive activation of the brain during rest likely reflects its involvement in self-generated cognitive activity and internal monitoring processes (Meyer and Lieberman, 2018; Schneider et al., 2008). Both DLPFC and lIFG are key components of the executive control system, which remains active at baseline to support internal cognitive organization and maintain readiness for external demands. This form of “default activation” has been well documented in both fNIRS and fMRI research, showing higher prefrontal oxygenation and metabolism during rest compared to low-demand or passive conditions (Mars et al., 2012; Matsuda and Hiraki, 2006; Xie et al., 2024). Such increased resting activity has been interpreted as reflecting a preparatory or readiness function in previous studies, although our data do not directly test this assumption. The observed activation pattern (rest > conflict > neutral) suggests that engaging in tasks—whether conflictual or neutral—leads to cortical deactivation in the DLPFC and lIFG relative to rest (Koshino et al., 2023; Menon, 2023). When individuals shift to social or externally focused interactions, these prefrontal regions decrease activity, reflecting a switch from internally focused self-regulation to externally directed attention (Raichle et al., 2001; Buckner et al., 2008). For example, fNIRS studies show decreased activation in the anterior left PFC during role-playing tasks, indicating less engagement of self-referential and introspective networks when individuals adopt another's perspective or perform socially embedded behaviors (Lim et al., 2024b).

Furthermore, our analyses showed a distinctive activation profile marked by greater cortical deactivation during the neutral condition compared to the conflict condition. This finding aligns with those of Matsukawa and colleagues, who reported that the PFC showed a significant decrease in activation during neutral video viewing, indicating a broad pattern of deactivation. Conversely, viewing emotionally arousing clips, such as horror scenes (negative emotion), caused only minimal changes — that is, a mild or nonsignificant decrease. In other words, the neutral viewing condition triggered more prefrontal deactivation than the more arousing conflict or threat conditions (Matsukawa et al., 2018). This pattern might be related to the dynamic balance between the default mode network (DMN) and task-positive networks. Since the brain's networks operate in a push-pull manner, when the DMN is active during neutral or routine social engagement, the task-positive network (including DLPFC/IFG) tends to deactivate (Mancuso et al., 2022; Menon, 2023; Raichle et al., 2001). In contrast, conflict situations require sustained cognitive regulation and suppression of the DMN, leading to reduced deactivation in the DLPFC and IFG (Wittfoth et al., 2009).

During the active role-playing task in the present study, a similar distinction between the conflict and neutral conditions was also observed in the DLPFC and IFG. Overall, well-practiced neutral interactions, like polite role-playing, may suppress DLPFC and IFG activity below their default levels, as individuals rely on automated social scripts rather than effortful control (Weissman et al., 2008). Conversely, conflict scenarios likely engage additional social-cognitive processes—such as perspective-taking, mentalizing about others' intentions, or emotional appraisal—which can counteract the typical task-based deactivation. In fact, evidence shows that brain regions in the DMN can exhibit less suppression or even positive activation during emotionally charged or social tasks (Di Plinio et al., 2018). Although our study did not directly assess DMN activity, the observed pattern is consistent with prior findings suggesting that emotionally or socially demanding situations may maintain higher DMN engagement. Therefore, conflict trials did not suppress the “default” network as much, aligning with the observed pattern (Jack et al., 2013).

Interestingly, the rTPJ deviated from this general deactivation pattern, showing a weak trend toward positive activation during conflict in the active role-playing condition. The rTPJ is a key hub for mentalizing and perspective-taking—the ability to infer others' intentions and beliefs (Saxe et al., 2004; Schurz et al., 2014). For example, disrupting the rTPJ impairs one's ability to handle conflicting viewpoints and moral dilemmas, indicating its essential role in conflict resolution and theory-of-mind processes (Buckner et al., 2008; Qureshi et al., 2020; Schilbach et al., 2013). Its selective activation suggests that, although conflict generally reduces shared neural processing, it still activates localized social-cognitive mechanisms. In emotionally charged or competitive situations, individuals may try to interpret their partner's intentions or predict responses, temporarily engaging the rTPJ (Decety and Lamm, 2007). Consistent with previous hyperscanning studies, disagreement or conflicting goals often increase activity in the rTPJ and nearby temporal-parietal areas, indicating heightened perspective-taking despite overall cortical downregulation (Redcay and Schilbach, 2019). The divergence in rTPJ activity may indicate that interpersonal conflict engages perspective-processing networks more strongly than neutral interactions.

Crucially, our findings showed a significant decrease in IBS during interpersonal conflict compared to control conditions. IBS usually occurs when people constantly predict and adjust to each other's behavior in real time. During positive or cooperative interactions, partners make continuous mutual predictions—such as finishing each other's sentences or expecting each other's actions—which enhances neural coupling (Hoehl et al., 2021). In fact, when two individuals intentionally try to predict each other's movements or responses, their brains show greater synchrony (Kayhan et al., 2022; Zhang et al., 2024). Conversely, during interpersonal conflict, this predictive coordination breaks down. Conflictual conversations are often disjointed: people interrupt, talk over one another, or respond unexpectedly. This unpredictability—sometimes intentional, like offering an unanticipated rebuttal—disrupts the timing that would normally maintain neural alignment (Léné et al., 2021).

Furthermore, conflict often triggers different attention patterns and individual regulatory processes, such as self-directed emotion regulation and defensive cognitive strategies. In these moments, individuals tend to focus inward on managing their internal states—like forming counterarguments, suppressing impulses, or controlling negative feelings—rather than paying attention to their partner's cues (Morawetz et al., 2017). This inward focus and reliance on self-regulation weaken the shared mental models and joint attention frameworks that support neural synchronization. As a result, IBS decreases as social partners disengage from reciprocal prediction and co-regulation, indicating a breakdown in the dynamic connection of their cognitive and emotional systems (Hirsch et al., 2021; Liang et al., 2022). Therefore, lower inter-brain coupling during conflicts can be seen as a neural sign of the disconnection between interacting minds. Reduced synchrony in key social-cognitive and regulatory areas may show a shift from cooperative, mutually engaged processing to more isolated, self-focused thoughts and feelings—patterns that reflect interpersonal disagreement (Long et al., 2021). Additionally, the heightened emotional arousal and negative feelings typical of conflict increase the demand for internal emotion regulation and cognitive resources, making intrapersonal neural synchrony more prominent than interpersonal synchrony. This inward turn in cognitive–affective processing interrupts the neural mirroring and dynamic coupling usually seen during cooperative or emotionally tuned interactions (Balconi and Vanutelli, 2017).

Finally, it's also crucial to consider how conflict is induced—whether passively (by observing conflict) or actively (by engaging in conflict)—as this can influence the pattern of IBS changes. In passive viewing scenarios, two individuals might simultaneously watch a scene of interpersonal conflict, such as a video of a heated argument, vs. a non-conflict scene (Froese et al., 2024). Even though they are not arguing with each other, conflict content can still decrease the synchronicity of their brain responses compared to neutral content. This occurs because each viewer's personal reactions to the conflict may differ—one might empathize with a particular character or feel anxious, while the other remains detached or takes the opposite side. Their emotional and cognitive responses to the same video thus diverge, resulting in lower inter-brain coherence. In active role-playing or real interpersonal conflict, the effects on IBS can be deeper and more complex (Markus and Shamay-Tsoory, 2024). Here, the individuals are not just spectators; they are participants in the conflict. Active conflicts are likely to trigger stronger emotional and strategic engagement, which could contribute to the observed IBS patterns.

## 5 Limitations and implications

The present study recognizes several limitations that should be addressed in future research. First, the experimental design employed passive video-viewing and scripted role-playing paradigms to study interpersonal conflict. While these methods provided good experimental control, they may not fully capture the complexity, spontaneity, and dynamic interactions typical of naturally occurring interpersonal conflicts. Future research should include more ecologically valid interactive tasks, such as real-time, unscripted conflict interactions, to better reflect authentic social dynamics and reciprocal processes during conflict scenarios (Moffat et al., 2024). Second, in an effort to reduce potential gender-related confounds, this study only included same-gender dyads. However, the gender makeup of interacting individuals can significantly influence neural and behavioral responses during interpersonal conflict. Therefore, future studies should systematically include both same- and mixed-gender dyads to clarify the impact of gender interactions on neural synchronization and conflict processing (Lim et al., 2024a). Third, due to the technical limitations of the fNIRS method, our study was limited to a specific set of cortical regions. Although our chosen areas were theoretically justified, future research should aim for broader cortical coverage, including additional critical regions such as the medial prefrontal cortex and relevant subcortical structures. This expansion would enable a more comprehensive mapping of the neural networks involved in interpersonal conflict and deepen our understanding of the underlying cognitive and emotional mechanisms.

## 6 Conclusions

This study systematically explored the neural basis of interpersonal conflict by combining fNIRS hyperscanning with two different paradigms: passive video observation and active role-playing. The results showed an unexpected activation pattern—brain activity was highest at rest, lower during conflict, and lowest during neutral interactions. At the same time, conflict reduces neural synchronization between individuals, reflecting the loss of harmony in their interaction. These findings, supported by similar studies in social neuroscience, form a consistent picture: when people connect and cooperate, their brains synchronize and social networks activate; when they clash, that unity breaks down both behaviorally and neurally. Understanding this neural dynamic not only supports our intuitive feelings in conflict (such as feeling “out of sync”) but also could guide strategies for conflict resolution.

## Data Availability

The raw data supporting the conclusions of this article will be made available by the authors, without undue reservation.
